# The origin of brain malignancies at the blood–brain barrier

**DOI:** 10.1007/s00018-023-04934-1

**Published:** 2023-09-09

**Authors:** Brennan McDonald, Kathrin Barth, Mirko H. H. Schmidt

**Affiliations:** Institute of Anatomy, Medical Faculty Carl Gustav Carus, Technische Universität Dresden School of Medicine, Dresden, Germany

**Keywords:** Cancer, Brain metastasis, Tumor microenvironment, Organotropism, Pre-metastatic niche, Metastatic niche, Circulating tumor cells, Neurovascular unit, Extracellular matrix

## Abstract

Despite improvements in extracranial therapy, survival rate for patients suffering from brain metastases remains very poor. This is coupled with the incidence of brain metastases continuing to rise. In this review, we focus on core contributions of the blood–brain barrier to the origin of brain metastases. We first provide an overview of the structure and function of the blood–brain barrier under physiological conditions. Next, we discuss the emerging idea of a pre-metastatic niche, namely that secreted factors and extracellular vesicles from a primary tumor site are able to travel through the circulation and prime the neurovasculature for metastatic invasion. We then consider the neurotropic mechanisms that circulating tumor cells possess or develop that facilitate disruption of the blood–brain barrier and survival in the brain’s parenchyma. Finally, we compare and contrast brain metastases at the blood–brain barrier to the primary brain tumor, glioma, examining the process of vessel co-option that favors the survival and outgrowth of brain malignancies.

## Introduction

Brain metastases involve tumor cells from peripheral sites escaping their primary location, circulating in the blood, disrupting the blood–brain barrier (BBB), extravasating into the brain, and therein producing malignant secondary tumors [1]. Representing the majority of intracranial tumors, brain metastases occur frequently in patients with advanced malignancies, such as breast, lung, skin, prostate, ovarian, and colorectal cancer [2]. The occurrence of brain metastases is estimated at 10–40% in patients with solid malignant tumors, with the incidence of brain metastases having increased over the last several decades [2]. This increase is a product of both advances in brain tumor detection and improvements in systemic extracranial therapy, which by extending survival also increase the risk that brain metastases will eventually occur. Indeed, a significant number of cancer patients, free from brain metastases at initial diagnosis, will develop intracranial tumors during the course of their disease or, in some cases, years after treatment of the primary tumor [3, 4]. Importantly, brain metastases are particularly lethal, with a median survival estimated between ≤ 4 and ≤ 12 months across all species of primary tumor [5, 6]. A principle reason for this lethality is that despite improvements in extracranial chemotherapy, few successful chemotherapeutic options are available for brain metastases, with treatment often consisting of palliative local approaches such as stereotactic or large field radiation and neurosurgical resection.

A crucial aspect in the etiology of brain metastases is our understanding of how blood-borne circulating tumor cells (CTCs) are able to overcome the BBB. Clinically, it is observed that certain primary tumors preferentially metastasize to particular secondary sites, above and beyond what would be predicted by vessel connections and anatomical proximity [7]. This preferential metastatic colonization, known as organotropism, describes how CTCs possess attributes that allow them to act as *seeds* favored to become disseminated tumor cells (DTCs) at organ sites with the appropriate *soil*, that is, organ properties which favor metastatic invasion and development [8]. For example, of the primary tumors metastasizing to the brain and spinal cord, the majority of cases (67–80%) are made up of lung cancer, breast cancer, and melanoma [9]. For CTCs to extravasate into the brain they need properties which favor interaction with (and disruption of) the BBB. Additionally, the brain’s microenvironment requires DTCs to possess or develop characteristics that allow for survival in the unique parenchyma of the brain, significantly different from any peripheral site, including limited access to nutrients, region-specific immune processes, distinct resident cells, and hypoxic conditions. This process of colonization and survival often involves a bi-directional series of events: On the one hand, tumor cells are able to adapt to the microenvironment of the brain. On the other hand, tumor cells can also alter the surrounding extracellular matrix (ECM) and resident cells to form their own brain metastatic niche. Importantly, the vast majority of tumor cells that bridge the BBB fail to survive or grow macrometastases in the brain [10, 11], while those that do survive initially remain in the perivascular microenvironment [10]. This creates a profound selective pressure for those tumor cells that are best able to colonize the vasculature of the brain. In this review, we focus on core contributions of the BBB to the origin of brain malignancies. We first discuss the anatomy of the BBB under physiological conditions. We then turn our focus to the neurotropic mechanisms that tumors possess to facilitate survival and malignant outgrowth into the brain.

## The blood–brain barrier: selective gatekeeper of the neurovascular unit

**Fig. 1:**
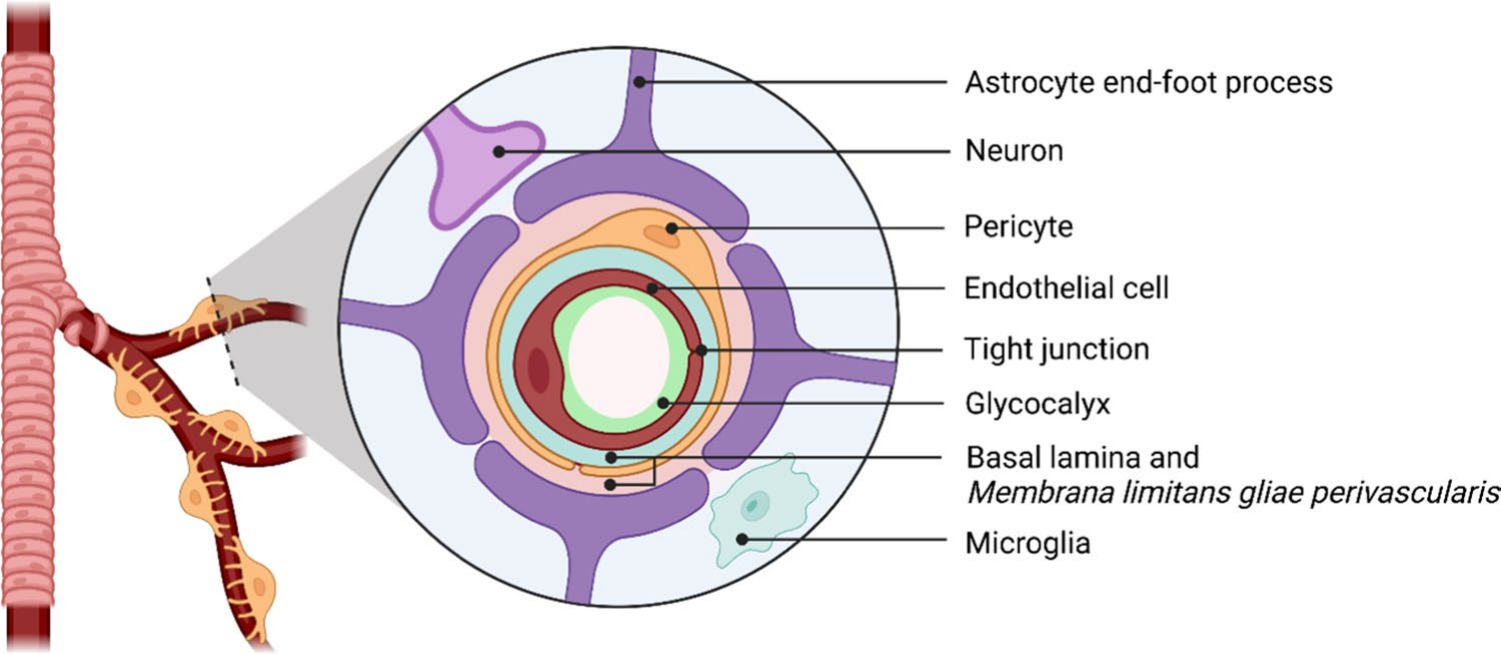
The blood–brain barrier of the neurovascular unit. The molecular (glycocalyx, basal lamina, *membrana limitans gliae perivascularis*) and cellular (endothelial cells, pericytes, astrocytes, neurons, microglia) components of the blood–brain barrier. The basal lamina (produced by endothelial cells and pericytes) and *membrana limitans gliae perivascularis* (produced by astrocytes) form a continuous extracellular matrix under physiological conditions. Created with BioRender.com

The neural networks of the CNS require that the parenchyma is constantly protected from toxins, pathogens, and ions leaking in via the circulating blood. At the same time, the brain is one of the most metabolically active organs in the body, receiving approximately 15–20% of the systemic cardiac output which provides a continuous supply of nutrients and energy substrates to maintain its activity. To achieve this delicate balance, the CNS has evolved a highly regulated, protective barrier between the blood vessel and brain tissue, the BBB (Fig. 1), which involves the complex structural and functional relationship between endothelial cells (ECs), pericytes, the neurovascular basal lamina (BL), and cells of the CNS (astrocytes, neurons, microglia, and perivascular macrophages). Indeed, the current literature discusses the BBB as a core anatomical and functional aspect of the neurovascular unit (NVU) [12]. The NVU describes the close developmental, anatomical, and functional relationships shared between the vasculature, cells of the CNS, and surrounding ECM. This includes their shared role in the regulation of the BBB and perivascular microenvironment, control of cerebral blood flow, and coordinated response to brain damage. The NVU, thus, describes the multifaceted responses of vascular and CNS cells acting as a unified biological interface.

### Endothelial cells

The BBB exists at all levels of the vascular tree within the CNS, including the penetrating arteries and arterioles, dense capillary bed, post-capillary venules, as well as draining venules and veins. Particularly important to BBB structure and function are ECs, modified simple squamous epithelial cells derived from the mesoderm and covering the vessel walls. In larger brain vessels, ECs form a cylindrical lattice of numerous, tightly bound individual cells. As one moves into the cerebral microvasculature, the diameter of the vessels decreases and the boundary of the lumen consists of only a few ECs, or a single EC folded upon itself, with a lumen diameter of approximately 7 µm. In the CNS, ECs form continuous capillaries (with the exception of the circumventricular organs) and are particularly thin, with less than 0.25 µm between the cell’s luminal and abluminal surface [13]. Despite this limited distance, neurovascular ECs manifest several core properties of the BBB, including a unique membrane composition, rich in various lipid species, which contribute to a pronounced negative charge [14]. Significantly reduced transcellular movement and vesicular transport is also observed on the luminal EC surface [15], as well as a high quantity of efflux transporters working to eject lipid soluble substances back into the blood [16]. In addition, low constitutive expression levels of intercellular adhesion molecule-1 (ICAM-1) on the luminal EC surface limits immune cell extravasation into the CNS [17]. Indeed, the brain’s immune surveillance under physiological condition is significantly less than in other tissues [18]. To facilitate the constant supply of oxygen required to power the brain, the lipid membrane of neurovascular ECs allows for the unrestricted diffusion of small gaseous molecules (e.g., O_2_, CO_2_). At the same time, specialized blood-to-brain influx transport mechanisms are in place to provide nutrients like glucose and amino acids that are unable to diffuse freely into the brain. The influx of ions and charged molecules is further tightly regulated to limit disruption of the functional circuits of the CNS. Altogether, neurovascular ECs represent the most significant cellular component of the BBB, generating the primary structural and functional barrier between the blood and brain’s parenchyma.

### Endothelial glycocalyx

Luminally, ECs are covered by a negatively charged layer, the endothelial glycocalyx, which is increasingly recognized as an important player in the structure and function of the BBB. Regarding chemical composition, the endothelial glycocalyx is principally composed of three main classes of molecule: proteoglycans, glycosaminoglycans (GAGs), and glycoproteins [19]. Proteoglycans consist of a core protein covalently bound to one or more GAG carbohydrate chains and exist as either membrane-bound (e.g., syndecans, glypican-1) or “free-floating” proteins (e.g., perlecan) suspended in the glycocalyx gel and capable of diffusing into the blood stream [20]. While a diverse array of membrane-associated proteoglycans are expressed on the EC luminal surface, the most prominent are the heparan sulfate proteoglycans (HSPG), specifically the syndecans and glypican-1. The structural and functional diversity of proteoglycans is extended by various combinations of the covalently attached GAGs (e.g., heparan sulfate), which are long, linear carbohydrate chains of repeating disaccharide subunits, which form dense branches extending out from the core protein. Another molecular component of the endothelial glycocalyx are membrane-bound glycoproteins, which are primarily cell adhesion molecules involved in intracellular signaling, inflammatory processes and immune cell extravasation. Similar to proteoglycans, a structurally and functionally diverse array of glycoproteins are present, including members of the selectin family, integrin family, and the immunoglobulin superfamily.

With respect to ultrastructure, the prevailing model argues that the endothelial glycocalyx is a bi-layer sugar–protein fiber matrix [21]. This bi-layer model describes two layers contributing to glycocalyx ultrastructure: (i) a thin (200–300 nm), stable inner layer forming a dense meshwork strongly anchoring to the EC membrane, and (ii) a much larger (460 nm–1 μm), robust but porous outer layer, consisting of mostly negatively charged GAGs and adsorbed plasma proteins, which is able to dynamically respond to the chemical and physical properties of the blood, including to shear stress and incorporating/exchanging components with the plasma.

From a functional perspective, the endothelial glycocalyx provides several contributions to the physiology of the BBB. It is an important regulator of vascular permeability and cell–cell interactions, with both the physical influence and negative charge of the glycocalyx limiting blood-borne cells and large molecules from perfusing across or interacting with the endothelial surface. Indeed, the large, dynamic outer layer of the glycocalyx (extending up to 1 μm into the lumen) works against the interaction of endothelial surface adhesion molecules and those of circulating cells (e.g., immune cells, tumor cells) by providing the surface adhesion molecules (extending out often only 10 nm) a physical and negatively charged shield, made up of GAGs, suspended proteoglycans, absorbed water, and plasma proteins. The endothelial glycocalyx is also involved in mechanotransduction and response to shear stress [22, 23], as well as contributing to anticoagulant pathways and fluid homeostasis [19], thus maintaining a steady-state between the blood and the vessel. Taken together, these properties of the endothelial glycocalyx provide a polar, chemical, and physical barrier on the luminal surface of ECs in the brain, regulating immune cell interactions, vascular permeability, and homeostasis which together enhance the functions of the BBB.

### Endothelial cell junctions

In addition to the glycocalyx, ECs have evolved highly specialized and enriched cell junctions, creating tight adhesions between the lateral membranes of adjacent ECs. The junctional complexes of ECs form from multiple transmembrane proteins, consisting of tight junctions (TJs) and adherens junctions (AJs), involved in restricting and regulating the paracellular flux of immune cells and solutes from the blood into the brain parenchyma. Based on the organization of TJs and AJs on the lateral surface of neurovascular EC, the paracellular region is separated into apical and basolateral domains. At the BBB, highly specialized and unique TJs are the most apical EC junctional protein complex, often located around the paracellular cleft. They form from tight junction-associated MARVEL proteins (in particular occludin and tricellulin), proteins of the claudin family (in particular, claudin-5 and claudin-12) and junctional adhesion molecules (JAMs). The extracellular domains of these proteins connect to one another through homophilic binding, allowing for the connection of two adjacent EC membranes. Additionally, unique tricellular TJ molecules such as LSR and MARVELD have been described where three adjacent ECs come into contact [24]. Inside the EC, TJ transmembrane structures link to zonula occludens (ZO) proteins, cytoplasmic plaque proteins acting as a scaffold, providing intracellular connections to the actin fiber cytoskeleton. Crucially, this scaffolding is dynamic, responding to mechanical and chemical signaling as well as the physiological environment of the EC, such as inflammation [25]. Contributing to the specialization of the BBB, TJs regulate the diffusion of ions and solutes down concentration gradients across the paracellular space, while also restricting the free movements of proteins and lipids from the luminal and abluminal EC membrane, generating high transendothelial electrical resistance [26]. Importantly, TJ disruption is associated with numerous disease states [33], leading to increased permeability [27]. In contrast to TJs, AJs are located at the basolateral EC membrane. AJs are composed of transmembrane cadherins (in particular, epithelial cadherin and vascular endothelial cadherin) and intercellular catenins connecting to the cytoskeleton, again via actin, with platelet and endothelial cell adhesion molecule-1 (PECAM1) critically involved in regulating AJ formation [28]. While the manifold functions of AJs are not fully understood, evidence suggest that unlike TJs, AJs are less involved in establishing a paracellular barrier but are involved in supporting vessel integrity by managing tensile forces acting on ECs. They also are believed to facilitate cell–cell contacts, respond to diverse signaling pathways, establish cell polarity, and promote the maturation, maintenance, and plasticity of TJs [29]. In regard to this latter function, increasing evidence suggests that complex crosstalk occurs between the components of AJs and TJs generating and regulating the endothelial paracellular barrier [29].

### Basal lamina

The abluminal surface of ECs is bound to the BL, a thin and selectively permeable membrane, formed by ECs and pericytes, adhering to these cells via integrins and enveloping the blood vessels and associated mural cells. The BL, measuring 50–200 nm, provides structural support, as well as creating an extracellular signaling interface for the cells of the NVU. With respect to the BBB, the BL provides an additional physical layer that resists the migration of cells and molecules towards—and separates ECs from—the brain’s tissue. Beyond the endothelial BL, a parenchymal boundary membrane termed the *membrana limitans gliae perivascularis* (MLGP) is intimate to the parenchyma of the brain. The MLGP is primarily produced by astrocyte end-feet processes, which tightly ensheath the vascular throughout the CNS, regulating and amplifying BBB properties [30, 31]. Under physiological conditions, these two extracellular membranes form an indistinguishable, continuous boundary, separated only by pericytes. However, a potential perivascular space exists between these two layers. In the case of pathology, leucocytes may congregate in this perivascular space, which acts as a regulatory checkpoint for further passage into the brain’s parenchyma [32, 33]. Regarding structure and composition, the BL is a highly organized, three-dimensional network primarily made of collagen IV proteins, nidogens, HSPG (in particular, perlecan and agrin) and laminins. The BL contains laminin-411 and laminin-511 derived from ECs, with low expression regions of laminin-511, acting as exit points for T-cell extravasation [32]. In larger penetrating arteries and arterioles, the MLGP includes laminin-111 (derived from pial cells) and laminin-211 (derived from astrocytes), whereas in the microvasculature laminin-111 is not present [34, 35]. Depending on physiological conditions, additional molecules are also found in the BL, including fibulins, fibronectin, various other collagen types, and thrombospondin [36]. Moreover, HSPGs are able to act as storage molecules, suspending growth factors and other bioactive compounds that can be released from the BL during vascular remodeling. Functionally, the BL is crucial to establishing the BBB, with knockout of HSPGs (perlecan/agrin) or collagen IV producing embryonic lethality [37, 38]. The BL and MLGP, thus, represent complex, dynamic ECM interfaces of the NVU which play a crucial role in generating and regulating the integrity of the BBB.

### Pericytes

Pericytes are mural cells of microvessels, found intimate to the endothelium and involved in numerous supporting functions throughout the vasculature. In the CNS, pericytes are located between the BL and MLGP, and are, thus, suspended in the ECM of the neurovasculature. Unlike peripheral pericytes, which derive from the mesoderm, pericytes in the CNS derive from the neural crest [39], with the ratio of pericytes to ECs in the neurovasculature significantly greater than in the periphery. For instance, while muscle tissue vessels have a ratio of 1:100 pericytes to ECs, and lung vessels a ratio of 1:10, in the CNS this is estimated to be between 1:1 and 1:3 [40]. Morphologically, pericytes are flattened cells which extended multiple elongated processes along the abluminal surface of the endothelium. However, because these cells are suspended in the abluminal ECM they are rarely in direct contact with the EC membrane, instead forming discrete cellular adhesions such as peg-and-socket junctions, mediated by the adhesion molecule N-cadherin [41] as well as adhesion plaques, gap junctions, and tight junctions facilitating communication with ECs [42]. Regarding function, pericytes are involved in supporting angiogenesis, ECM deposition, endothelial proliferation, immune cell regulation, and inflammatory processes, as well as responding to neural activity to control blood flow [43]. In this latter respect, pericytes play a similar role in the brain’s microcirculation to smooth muscle cells as they possess contractile elements able to control the vessel diameter [42, 44], with pericytes being actively relaxed by the release of signaling molecules including prostaglandin E2 and NO via the neurotransmitter glutamate [44]. Finally, pericytes play a critical role in regulating the development of the BBB through interactions with ECs, as well as in preserving the barriers functionality across the lifespan [43, 45]. For example, in mouse models in which pericytes are ablated, endothelial hyperplasia, abnormal vasculogenesis [46], and increased BBB permeability are observed [45]. Overall, pericytes are a crucial supporting element in the homeostasis of the brain’s microcirculation, regulating numerous processes involved in BBB function and maintenance.

### Astrocytes

Astrocytes, the most abundant cell type in the brain, are glial cells that extend end-foot membrane processes, involved in forming the MLGP*.* The polarized end-foot processes of astrocytes almost completely ensheath the EC layer, basal lamina, and associated pericytes. Limited gaps found between the end-foot processes allow for the contact of neuronal synapses directly with the MLGP, providing for neuronal communication with the blood vessels, which contributes to blood-flow regulation and BBB permeability [47–49]. Astrocyte end-foot processes are unique from the deeper, parenchymal portions of the cell in contact with neurons and other glia, possessing specific properties associated with the MLGP and BBB. These include dystroglycan–dystrophin complexes tethering the end-foot process to the MLGP [50], gap/tight junctions connecting adjacent processes [51], as well as region-specific K + (K_ir_ 4.1 [52]), glucose (GLU1, [53]), and water channels (aquaporin-4, [54]), which are fundamental in maintaining the energy and ionic homeostasis of the brain’s perivascular microenvironment. Astrocytes are also implicated in regulating various signaling pathways associated with the BBB, monitoring innate immunity, as well as maintaining endothelial junctional complexes [55]. Astrocytes thus contribute to a unique covering of the vasculature found throughout the CNS, providing an additional protective and regulatory layer to the BBB.

### Microglia

Microglia, the resident immune cells of the CNS, are myeloid cells [56] that derive from hematopoietic precursors migrating from the yolk sac into the CNS during development [57]. Making up approximately 10–15% of the total cells in the CNS [58], microglia play a vital role in the innate immunity of the brain and spinal cord. Structurally, microglia are small (5–10 μm) cells that extend radial processes into the ECM of the parenchyma. Functionally, the processes of microglia sense for pathogens and toxins, with these cells responsible for antigen presentation and neuronal development under physiological conditions [59]. In the case of pathology (e.g., infection, tissue damage), a prominent model argues that microglia are able to transition to two activated phenotypes with divergent functions, termed M1 and M2, which involve either pro-inflammatory/pro-killing (M1 phenotype) or immunosuppression and neural repair (M2 phenotype) functions [60]. However, this polarized differentiation during pathology has been challenged, suggesting that these functions represent a continuum rather than a dichotomy [61]. Regardless, the core aspect of microglia during brain trauma is a broad range of activated functions related to immunity and repair in the CNS. At the BBB, microglial end-feet extend and connect with the MLGP and, along with astrocytes, form part of the glial ensheathment of the neurovasculature [62]. Microglia are implicated in a host of consequences at the BBB in numerous disease states resulting from inflammatory processes including multiple sclerosis, Alzheimer’s disease, and ischemic stroke [63]. Specifically, microglia activation is associated with the release of pro-inflammatory factors that influence the permeability of the BBB by producing alterations in the integrity of endothelial junctional complexes [64]. Importantly, along with the innate immunity of the CNS, peripheral immune cells are also directly involved in the regulation and permeability of the BBB both in health and disease [65, 66]. Indeed, crosstalk between microglia and peripheral immune cells play an important role in neuroinflammatory processes, influencing the leakiness of the barrier [66].

## Influence before invasion? Evidence suggesting a pre-metastatic niche at the blood–brain barrier

Important to our understanding of metastases is the realization that the microenvironment of distal organs play a profound role in the successful invasion and progression of CTCs. Indeed, the concept of the metastatic niche describes the alterations that occur in an organs microenvironment in the presence of successful DTCs, favoring the survival and progression of the metastatic lesion. More recently, growing evidence indicates that microenvironmental changes are able to occur even before CTCs arrive in distal organs, giving rise to the idea of a pre-metastatic niche. The concept of a pre-metastatic niche describes how primary tumors are able to actively and selectively modify a distal organ’s microenvironment through the release of factors and/or EVs, occurring before metastatic spread [67]. Put another way, the pre-metastatic niche describes the stepwise, complex molecular and cellular changes at a secondary site, induced by secreted or shedded factors by a primary tumor, prior to dissemination. Through this process, primary tumors gain the ability of *action at a distance*, priming secondary organs for metastatic invasion before CTCs arrive. Such changes in a distal organ microenvironment include disruption of the vascular barrier, alteration of local resident cells, ECM remodeling, release of pro-metastatic factors (e.g., growth factors, cytokines, chemokines), and alterations to immune function [67, 68]. Importantly, the pre-metastatic and metastatic niches both occur in the initial stages of metastatic development and although both niches conceptually follow one another, it is probable that their development and influence can occur both together and independently. As highlighted by Geissler and colleagues [69], the distinction between these niches is best viewed as a difference in function, with the pre-metastatic niche aiding in CTC access, anchorage, and early survival, while the metastatic niche promotes the survival, protection, and proliferation of DTCs. Pre-metastatic niche formation and its influence on metastatic invasion is described in detail for the liver [70], bone [71], lung [72], and lymph nodes [73]. However, far less is currently understood regarding the pre-metastatic niche at the BBB.

While findings are limited as to the exact nature of a pre-metastatic niche at the BBB, growing evidence suggest that such a priming event may occur in certain cancers. Recently, focus has been placed on tumor-derived EVs, in particular exomes, which have been demonstrated to shape the pre-metastatic niche of various organs [74–76]. Exosomes are tiny vesicles (50–100 nm in diameter) released by cells, able to contain small bioactive molecules, often contributing to paracrine signaling. However, exomes are also able to be shuttled through the blood and be taken up by distal cells. One such exome cargo molecule are microRNAs (miRs), which are tiny, non-coding stands of RNA, able to modulate gene expression [77].

With respect to the development of a brain pre-metastatic niche, exosome-mediated transfer of cancer-secreted miR-105 has been shown to suppress the tight junction protein ZO-1, is detected in the circulation at the pre-metastatic stage, and is associated with metastatic progression in breast cancer [78]. In a similar vein, exome-delivered miR-181c is demonstrated to facilitate disruption of the BBB both in vitro and in vivo by downregulating the gene PDPK1, which causes abnormal localization of actin [79]. Interestingly, Tominaga and colleagues [79] suggest that tumor-derived exomes likely contain multiple miRs that each alter and disrupt the BBB through different mechanisms, thus facilitating tumor migration. Long non-coding RNAs are also implicated in this processes, with Xu and colleagues [80] demonstrating that EV-delivered LINC00482 inhibits miR-142-3p in the perivascular microenvironment, upregulating the expression of TGF-β1 which promote microglial M2 polarization in brain metastatic lung cancer, thus contributing to the pre-metastatic niche in vivo. Hoshino and colleagues also observed that exomes isolated from organ-specific metastatic breast cancer cells travel exclusively to the associated organs in vivo [75]. This included exomes obtained from brain metastatic breast cancer cells traveling to the BBB, in this case with PECAM1-positive brain ECs representing 98% of the exome-containing cells [75]. Moreover, they show that the adhesion molecule integrin β_3_ was significantly expressed on those exomes that preferentially accumulated in the brain. These results, thus, suggest not only that exomes are able to possess integrin β_3_ facilitated neurotropic specification but also that the endothelium of the BBB plays an important role in the establishment of a brain pre-metastatic niche.

Finally, in addition to exomes, freely circulating miR-122 secreted by breast cancer cells has been shown to downregulate the glycolytic enzyme pyruvate kinase, suppressing glucose uptake by astrocytes, both in vitro and in vivo [81]. Moreover, inhibition of miR-122 in vivo restored glucose uptake in the brain, reducing the incidence of metastasis [81]. Given the importance of glucose metabolism for metastatic tumor cell survival and growth, these results suggest that an aspect of brain pre-metastatic niche formation may involve alterations in resident cell energy utilization to favor tumor cell access to energy substrates, facilitating progression. Thus, when taken together, emerging evidence suggests that alterations BBB and perivascular microenvironment through tumor-secreted factors may favor the creation of a pre-metastatic niche in the brain. In turn, such a niche has the potential to assist CTC invasion. Ultimately, the development of future techniques to experimentally define [69] and identify the development of a pre-metastatic niche in the brain could allow preemptive interventions to mitigate brain metastases [67].

## Breaking and altering: mechanisms facilitating tumor cell extravasation and metastatic niche formation

**Fig. 2:**
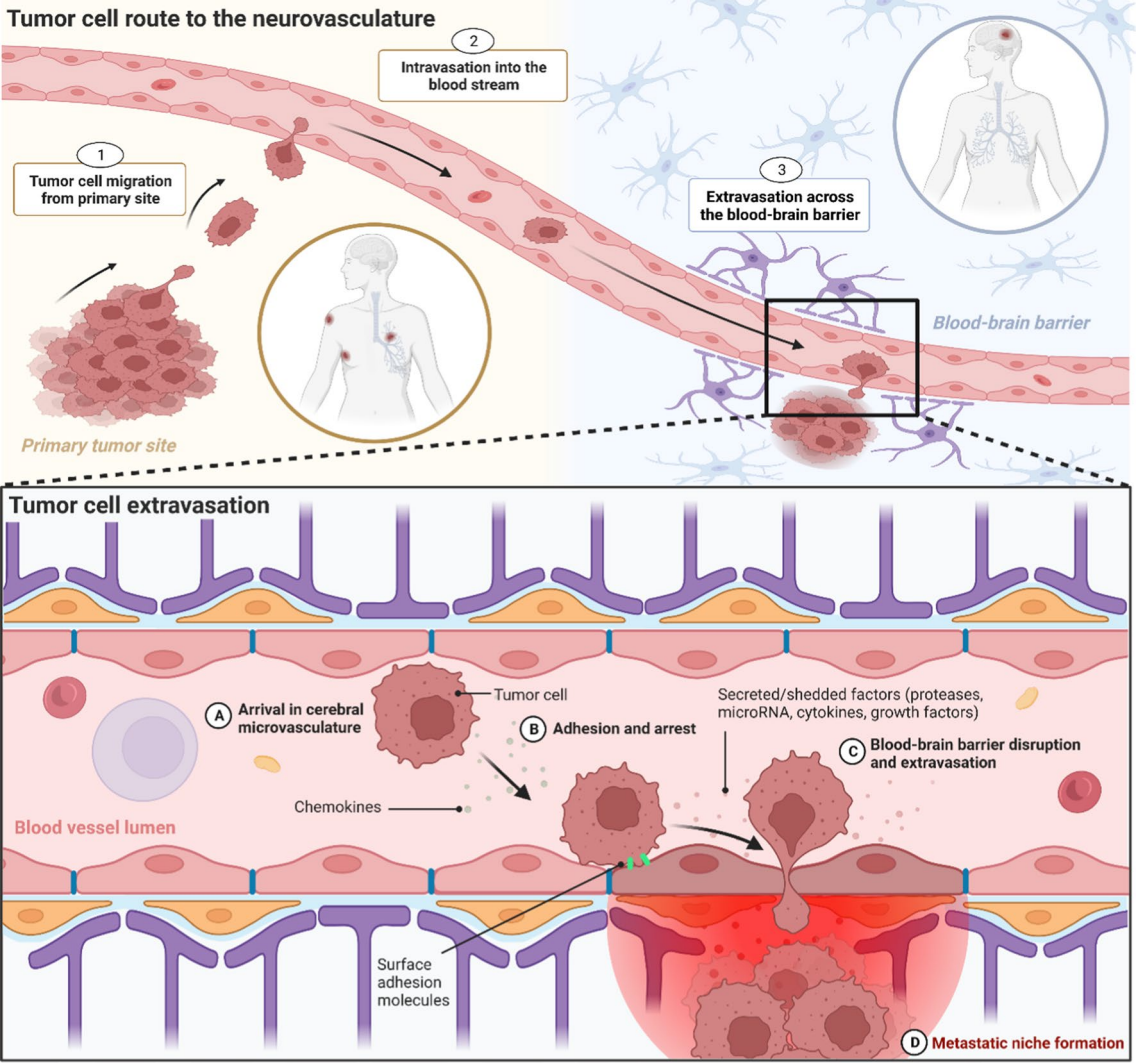
The origin of brain metastases at the blood–brain barrier. *Tumor cell route to the neurovasculature.* **1** Tumor cells at the primary site accumulate attributes that enable the manipulation of the surrounding tissue, allowing for cell migration and tissue invasion. **2** Tumor cells reach blood vessels at the primary site and intravasate across the vessel wall into the blood stream. **3** Circulating tumor cells reach the neurovasculature, arrest on the endothelium and extravasate across the blood–brain barrier. *Tumor cell extravasation.* **A** Tumor cells arrive in the neurovasculature, where chemokines are able to facilitate attraction to the brain endothelium. **B** Arrest on the brain endothelium is supported by vessel dynamics, as well as surface adhesion molecules located on both the endothelial and tumor cell membrane. **C** Various secreted and shedded factors released by the tumor cell disrupt the blood–brain barrier and facilitate extravasation across the brain endothelium. **D** After extravasation, disseminated tumor cells remain in the perivascular microenvironment where they continue to release factors that support the development of a metastatic niche. Created with BioRender.com

Before extravasation across the BBB can occur, tumor cells at a primary site must develop to a stage whereby they are able to exfiltrate their original location, enter the blood stream, and reach the brain. Core features of this development include (i) undergoing epithelial-to-mesenchymal transition (EMT) which enhances aggressiveness and stem-like characteristics, (ii) infiltrating and manipulating the ECM of the primary site, and (iii) intravasating into the blood steam. This path from primary tumor to brain metastases is a complex, multistep process, described in detail elsewhere [10, 82, 83]. Here, we focus on the mechanisms facilitating tumor cell extravasation across the BBB (see Fig. 2). The extravasation of tumor cells across the EC layer within the brain occurs predominantly in capillaries and post-capillary venules, particularly at capillary branches [10, 84]. As the lumen diameter is smallest at these locations, this maximizes the force of blood flow, which is able to distort and flatten CTCs against ECs and the associated glycocalyx, increasing the potential for arrest and adhesion [85, 86]. Once arrest has occurred, tumor cells have two potential means of extravasating across the BBB. First, the paracellular route between the lateral membranes of ECs or second, the transcellular route, migrating through the EC by establishing a transcellular pore [87]. Importantly, extravasation across the endothelium is a rate-limiting step in the development of brain metastases. In particular, arrest of CTCs along the neurovasculature is a non-trivial process, requiring specific mechanisms to facilitate adhesion and transmigration. Indeed, an important aspect of our understanding of metastatic development is the finding that a vast number of CTCs are often observed in patients’ blood in comparison to the actual number of extra- or intracranial metastatic lesions that develop [88]. Metastatic spread from a primary to a secondary site, therefore, appears to be a highly inefficient process, as few CTCs are ever able to establish metastases, including crossing the BBB and colonizing the brain [89]. This suggests that those CTCs that are able to successfully detect, arrest, and then cross the BBB must possess mechanisms that favor this series of events. In support of this, growing evidence indicates that neurotropic tumor cells display genetic changes that correlate with brain invasion. For example, patient-derived brain metastatic lesions across several cancers have been shown to possess mutations not observed in matched primary tumors or extracranial metastases [90]. However, within individuals, genetic homogeneity is observed across metastatic brain lesions regardless of spatial distribution or temporal onset, suggesting a conservation of neurotropic properties once they arise in metastatic cells [90]. Moreover, within different primary tumor molecular subtypes, neurotropic specificity also exists, with certain genetic changes enhancing dissemination into the brain. For example, brain metastatic triple-negative or basal-type breast cancers disrupt the BBB and colonize the brain, whereas BBB permeability remains unaltered by HER2/neu-positive breast cancer [91]. In addition, CNS metastases develop in around half of patients with mutant epidermal growth factor receptor (EGFR) or anaplastic lymphoma kinase-rearranged non-small-cell lung cancer, suggesting these genetic alterations are involved in neurotropic secondary tumors [92].

In a similar vein, how the brain is colonized appears to be influenced by tumor cell molecular subtype. For example, the molecular subtype of brain metastatic lung cancer has a pronounced influence on the spatial distribution of metastatic lesions in the cranial cavity [93]. Moreover, recent work by Basnet and colleagues indicates that genetic adaptation to specific organ microenvironments occurs in tumor cells by demonstrating different patterns of gene activity between metastasis locations using in situ transcriptomic profiling [94]. Specifically, using mouse xenograft breast cancer micrometastases, they show brain-specific and lung-specific transcriptome signatures in secondary tumors, differing substantially both from each another and from the initial tumor cell population. They also demonstrate that the brain metastatic variant had reduced oxidative stress and antioxidative response, suggesting alterations favoring survival in the hypoxic microenvironment of the brain.

Taken together, these findings suggest that those tumor cells capable of achieving early-stage metastatic extravasation into and colonization of the brain undergo specific changes in their genetic profile. These changes likely promote mechanisms that favor neurotropism, not only facilitating disruption of the BBB but also the capacity to survive in the nutrient-sparse and hypoxic conditions of the brain parenchyma. In the following sections, we focus on several prominent neurotropic molecular mechanisms (in particular, secreted factors and cell-surface molecules) that tumor cells have at their disposal to manipulate BBB permeability, facilitate extravasation, and form a metastatic niche in the brain.

### Proteases

Various proteolytic enzymes are implicated in the formation of brain metastases by disrupting or remodeling the endothelial glycocalyx, junctional complexes and ECM of the BBB, thus facilitating tumor cell extravasation and survival (Table 1). To date, heparanase, an endoglycosidase able to cleave heparan sulfate from HSPGs, is one of the most investigated proteases involved in cancer, correlating with tumor angiogenesis, metastasis, and reduced survival across numerous cancers in various organ systems [95]. Regarding brain metastases, heparanase is implicated in the origin and proliferation of brain lesions arising from breast cancer [96–99] and melanoma [100–107]. With respect to mechanism, heparanase is involved in the proteolytic degradation of HSPGs at the BBB [104], leading to barrier disruption and ECM remodeling, which enhances tumor cell invasion of the brain. In addition, degradation of the ECM by cleavage of heparan sulfate has the potential to induce the release of numerous suspended factors, including growth factors, chemokines and other bioactive compounds that may further promote metastatic niche formation and cancer outgrowth [95]. Recent evidence also implicates heparanase in the regulation of angiogenesis [95], transcription [108], signaling pathways [109], and exosome generation [110], each of which may additionally enhance the invasion and survival of brain metastatic tumor cells. Given the abundance of HSPGs in the neurovascular glycocalyx, we further speculate that significant cleavage and sheading of heparan sulfate at the EC luminal surface may degrade glycocalyx integrity, potentially exposing underlying cell adhesion molecules, facilitating tumor cell extravasation.

**Table 1:** Proteases associated with tumor cell extravasation across the blood–brain barrier and the formation of brain metastases

Proteases	Findings	Primary tumor	Refs
ADAM8	Regulates MMP9 expression in tumor cells, with inhibition leading to reduced trans-endothelial migration in an in vitro BBB model	Breast cancer	[128]
ADAM9	ADAM9 expression is greater in highly brain metastatic tumor cells compared to bone-metastatic or primary tumor cells, with more invasive potential, increased adhesion capacities, and greater expression of α_3_β_1_ integrin	Lung cancer	[127]
Cathepsin S	Associated with decreased brain metastasis-free survival. Produced by macrophages and tumor cells. Facilitates BBB extravasation through proteolytic degradation JAM-B. Depletion via inhibitors significantly reduces brain metastasis in vivo	Breast cancer	[126]
Heparanase	Correlates with the brain metastatic potential of tumor cells in patients	Breast cancer	[98]
	EGFR-triggered nucleolar localization of heparanase produces DNA topoisomerase-I modulation and enhances brain metastatic proliferation		[97]
	Active and inactive heparanase enhances EGFR phosphorylation via Src, increasing tumor migration and proliferation, correlating with head and neck tumor progression		[96]
	Downregulation of miR-1258, involved in the expression of heparanase, inversely correlates with metastatic invasion to the brain		[99]
	Accumulates around blood vessels in brain metastatic melanoma specimens	Melanoma	[100]
	Increases the invasion potential of tumor cells across an in vitro BBB model		[101]
	Overexpression of the neurotrophin receptor p75^NTR^ on tumor cell surface links to the invasive properties of heparanase		[102, 103, 105, 106]
	Supra-additive levels detected when brain metastatic tumor cells were incubated with endothelial cells and astrocytes		[104, 107]
MMPs	Increased MMP1 expression in early circulating tumor cells with EMT phenotype	Breast cancer	[118]
	Higher expression of MMP1 and MMP9 in brain-metastasizing tumor cells compared to primary and bone-metastasizing tumor cells		[119]
	MMP1 was part of specific gene expression signature for brain (and lung) metastatic relapse compared to relapse associated with bone, liver or lymph nodes		[120]
	Strong upregulation of MMP9 observed in reactive astrocytes localized in the immediate vicinity of extravasated tumor cells		[84]
	MMP1 degrades tight junctions of the BBB. Ectopic expression of MMP1 increases the neurotropic potential of tumor cells not associated with brain metastases		[121]
	EMT-promoting transcription factor Slug enhances MMP1 expression via directly binding to the promoter region on tumor cells		[122]
	Targeted knockdown of MMP1 in mice attenuated brain and lung metastasis formation in vitro and in vivo		[114, 121]
	Elevated serum level of MMP9 (but not MMP2) in patients with brain metastases. Both MMP2 and MMP9 significantly increased in patients’ cerebrospinal fluid	Multiple types	[115, 116]
	Elevated levels of the aldo–keto reductase AKR1B10 is associated with MMP2 and MMP9 expression via MEK/ERK signaling, facilitating BBB TJ degradation in vitro. Silencing of AKR1B10 downregulated MMP2 and MMP9 expression, suppressing both in vitro and in vivo tumor cell extravasation across the BBB	Lung cancer	[113]
	Invasion of the brain parenchyma by tumor cells is associated with MMP2 and MMP9. Tumor cell influence on microglia morphology induce the release of MMP2	Melanoma	[123–125]
Serine protease	Facilitates extravasation across an in vitro BBB model by disrupting junction complexes and causing apoptosis in ECs. The use of a serine protease inhibitor approximately halved the number of tumor cells able to migrate across an endothelial monolayer	Melanoma	[129]

In addition to heparanase, matrix metalloproteases (MMPs) are also implicated in the disruption of the BBB during the occurrence of brain metastases. MMPs are a class of calcium-dependent, zinc-containing endopeptidases able to hydrolyze and breakdown components of junctional complexes and the ECM [111]. Because of the widespread proteolytic function of MMPs, they are implicated in the formation and promotion of the tumor microenvironment [112]. For example, in brain metastatic lung cancer cells, significantly elevated levels of the aldo–keto reductase AKR1B10 are associated with MMP2 and MMP9 expression via MEK/ERK signaling, facilitating TJ degradation in vitro [113]. Silencing of AKR1B10 in these tumor cells downregulated MMP2 and MMP9 expression, suppressing both in vitro and in vivo tumor cell extravasation across the BBB [114]. Clinically, in patients with brain metastases, elevated serum levels of MMP9 (but not MMP2) has been observed [115], while both MMP2 and MMP9 were significantly increased in patients’ cerebrospinal fluid [116]. Experimental data further suggest that astrocyte activity is likely involved in the expression of MMP2 and MMP9 during brain metastases [84, 117]. MMP1 is also heavily implicated in the development of brain metastatic breast cancer [114, 118–122], being associated with both degradation of BBB TJs and brain-metastasizing potential. Recent studies also highlight the role of MMP2 and MMP9 in brain metastatic melanoma [123–125], while targeted knockdown of MMP1 has been shown to attenuate brain and lung metastasis formation in vitro and in vivo [114, 121].

Finally, several additional proteolytic enzymes are implicated in the formation of brain metastases. In patients with primary breast tumors, high levels of cathepsin S, a member of the cysteine cathepsin protease family, is associated with decreased brain metastasis-free survival. Cathepsin S is produced by both macrophages and tumor cells and facilitates BBB extravasation through proteolytic degradation of the junctional protein JAM-B, with depletion of Cathepsin S via inhibitors significantly reducing brain metastasis in vivo [126]. In non-small-cell lung cancer, ADAM9 (a member of the “a disintegrin and metalloprotease” family) expression was shown to be significantly greater in highly brain metastatic tumor cells compared to bone-metastatic or primary tumor cells, with higher invasive potential, increased adhesion capacities, and greater expression of integrin α_3_β_1_ [127]. Additionally, in breast cancer ADAM8 is implicated in regulating the expression of MMP9, with inhibition leading to reduced trans-endothelial migration [128]. Lastly, serine proteases in melanoma have been demonstrated to facilitate extravasation across an in vitro BBB model by disrupting junction complexes and causing apoptosis in ECs. Interestingly, the use of a serine protease inhibitor approximately halved the number of melanoma cells able to migrate across an endothelial monolayer [129]. Taken together, numerous cancers associated with brain metastases, including breast, lung and skin cancer, are associated with the release of proteolytic enzymes, which degrade and remodel the junctional complexes and ECM of the BBB, facilitating extravasation and metastatic niche formation in the brain.

### MicroRNAs

MiRs, small non-coding RNAs involved in the post-transcriptional control of gene expression [130], are implicated in the formation of brain metastases (Table 2). Regarding miRs there are two promising avenues of investigation. First, miRs (either directly secreted or packaged in exomes) are implicated in mechanisms associated with BBB disruption and microenvironment alterations by tumor cells, representing a clinical target. Second, analysis of miR expression patterns in patients’ serum, cerebrospinal fluid, or tumor tissue, may offer a means of improving prognostic and diagnostic accuracy of brain metastases, acting as a potential biomarker. In both cases, miRs may be either upregulated or downregulated. Depending on the miR, this may either facilitate or mitigate a cancer’s metastatic potential.

**Table 2:** MicroRNAs associated with tumor cell extravasation across the blood–brain barrier and the formation of brain metastases

microRNAs	Findings	Primary tumor	Refs
miR-509	Highly expressed in primary tumors, while significantly downregulated in brain metastatic lesions. Regulates two genes: i) RhoC involved in MMP9 expression influencing cancer cell invasion and ii) TNF-α which modifies BBB permeability	Breast cancer	[132]
miR-7	miR profile analysis of cancer stem-like cells revealed that significantly lower level of miR-7 was related to preferential organotropism for the brain, with reduced miR-7 producing greater levels of Kruppel-like factor 4		[131]
miR-105	Identified in the circulation in pre-metastatic cancer patients, reflecting metastatic progression. Suppresses ZO-1, disrupting BBB integrity. Overexpression in non-metastatic tumor cells increases vascular permeability and brain metastases. Inhibition in highly brain metastatic cancer limits metastatic potential. Exosome-mediated miR-105 expression by brain metastatic breast cancer cells is associated with the reprograming of activated microglia, upregulating immune-suppressive cytokines and supporting metastatic niche formation		[78, 133]
miR-19a	Astrocyte-derived exosomes containing miR-19a targeting PTEN in brain metastatic breast cancer cells, which activates the PI3K/Akt pathway, promoting invasion of the brain parenchyma		[134]
miR-181c	Exome-delivered miR-181c facilitates disruption of the BBB in vitro and in vivo by downregulating the gene PDPK1, causing abnormal localization of actin		[79]
miR-122	Freely circulating miR-122 secreted by tumor cells downregulates the glycolytic enzyme pyruvate kinase, suppressing glucose uptake by astrocytes, in vitro and in vivo*.* Inhibition of miR-122 in vivo restored glucose uptake in the brain, reducing the incidence of metastasis		[81]
miR-1290 and miR-1246	High levels of tumor-secreted EV-mediated miR-1290 and miR-1246, activating astrocytes. Higher circulating EV levels in patients with metastases than without. MiR-1290- or miR-1246-overexpressing astrocytes promote mammospheres. Astrocytes overexpressing miR-1290, but not miR-1246, increase brain colonization and growth of tumor cells		[135]
miR-378	Overexpressed in both primary tumor and associated brain lesions compared to non-brain-metastasizing variants	Lung cancer	[137]
miR-328 and miR-330-3p	Expression pattern able to differentially predict patients positive and negative for brain metastases		[136]
miR-142-3p	EV shuttling of long non-coding RNA LINC00482 to microglia induces microglial M2 polarization by binding to miR-142-3p and upregulating TGF-β1. This in turn facilitates pre-metastatic niche formation in vivo		[80]
miR-150-5p, miR-15b-5p, miR-16-5p, and miR-374b-3p	Identified as a prognostic signature in a retrospective, cohort-based study analyzing genome-wide and targeted miR expression in primary melanoma tissue, improving predictions of brain metastases development	Melanoma	[138]

Several miRs are identified in influencing the integrity of the BBB and metastatic niche formation in brain metastatic breast cancer [78, 79, 81, 131–135], lung cancer [80, 136, 137], and melanoma [138]. For example, a miR profile analysis of cancer stem-like cells derived from breast cancer revealed that significantly lower level of miR-7 was related to preferential organotropism for the brain, with reduced MiR-7 producing greater levels of Kruppel-like factor 4 [131]. Also in breast cancer, exosome-mediated miR-105 expression by brain metastatic tumor cells is associated with the reprograming of activated microglia, upregulating immune-suppressive cytokines and supporting brain metastatic niche formation [133]. In addition, MiRs derived from resident cells of the brain are also implicated in metastatic niche progression [134, 135]. For example, astrocyte-derived exosomes containing miR-19a target PTEN in brain metastatic breast cancer cells, activating the PI3K/Akt pathway, promoting invasion into the brain parenchyma [134].

Regarding prognosis and diagnostics, the landscape of miRs at the primary tumor site or circulating in the blood may allow for the mapping of specific signatures, which improve metastasis identification and risk assessment [139]. For example, in melanoma, miRs expression exhibits a high frequency of genetic modifications [140], as well as melanoma-specific patterns [141]. Regarding brain metastatic melanoma, a retrospective, cohort-based study analyzing genome-wide and targeted miR expression in primary melanoma tissue identified a prognostic signature of 4-miR (miR-150-5p, miR-15b-5p, miR-16-5p, and miR-374b-3p) that improved predictions for the development of brain metastasis [138]. Moreover, miR-150-5p was shown to predominantly occur from tumor-infiltrating lymphocytes, suggesting that the immune factors are also a marker for patient outcomes.

Overall, while there is limited overlap between the miRs in these findings, it is important to bear in mind that these are early investigations into the prognostic and diagnostic significance of miRs in brain metastases. The goal of future work will be to validate, extend, and specify these results. In doing so, specific patters of miRs may prove a useful prognostic and diagnostic tool in the identification of primary tumors with the potential to metastasize to the brain.

### Growth factors

Growth factors are a superfamily of molecules, which are capable of promoting a diverse range of cellular processes related to growth and development. These include crucial roles in controlling cell proliferation, migration, and differentiation. Importantly, growth factors and their signaling pathways are implicated in the onset and progression of brain metastases across various cancers (Table 3). For example, hepatocyte growth factor (HGF) and angiopoietin-2 are involved in brain metastatic breast cancer [142, 143]; while HGF, placental growth factor (PLGF), and VEGF-A are associated with brain metastatic lung cancer [10, 144, 145]. Strongly implicated in brain metastases are a host of cytokines, shown to be involved in tumor dissemination across the BBB and metastatic outgrowth. Chemokines (or chemotactic cytokines) are an important subfamily of cytokines involved in stimulating the migratory behavior of leucocytes and are also implicated in attracting tumor cells to the brain endothelium. Importantly, cytokines and chemokines are also involved in promoting neuroinflammation, facilitating disruption of the BBB, altering immune cell behavior, and aiding the outgrowth of metastatic tumor cells into the brain parenchyma. Chemokines and cytokines are implicated in the formation of brain metastatic breast cancer [121, 146–153], lung cancer [80, 154–162], melanoma [123, 124, 163–167], and renal cell cancer [168]. For example, in brain metastatic breast cancer, Curtaz and colleagues demonstrated that BBB permeability in vitro was significantly increased after applying sera from breast cancer patients with brain metastases, but was not increased with sera from patients with bone or visceral metastases. Significantly increased levels of the chemokines CX3CL1 and CXCL13 were only detected in the sera from the brain metastatic breast cancer patients, which correlated with the tumor’s estrogen/progesterone receptor status [150]. Also in breast cancer, Chung and colleagues demonstrated that brain metastases-associated fibroblasts express significantly higher levels of CXCL12 and CXCL16 than fibroblasts from primary tumors or normal breast tissue, which increases tumor cell migration, with inhibition of CXCR4 or CXCL16 reducing tumor cell migration [147]. Moreover, recent findings indicate that resident cells of the CNS are also influenced by growth factors [121, 142, 146, 148, 153, 154, 166, 168–171]. In particular, several studies suggest that brain metastatic tumor cells are able to induce polarization of microglia towards a M2 (immunosuppressive) phenotype, increasing immune invasion and enhancing metastatic outgrowth [152, 158, 159, 172]. Taken together, a range of growth factors are implicated in the origin of brain metastases at the neurovasculature, influencing both the integrity of the BBB and manipulating the function of the immune system to create a more favorable metastatic niche.

**Table 3:** Growth factors associated with tumor cell extravasation across the blood–brain barrier and the formation of brain metastases

Growth factors	Findings	Primary tumor	Refs
CCL7	Brain-metastasizing tumor cells secrete MMP1 and COX-2, inducing CCL7 expression by activated astrocytes. This promotes BBB permeability and the formation of brain metastasis in vivo	Breast cancer	[121]
CX3CL1 and CXCL13	In vitro BBB permeability increases after applying sera from breast cancer patients with brain metastases, but was not increased with sera from patients with bone or visceral metastases. Significantly increased levels of the chemokines CX3CL1 and CXCL13 were only detected in the sera from the brain metastatic sera, which correlated with the tumor’s estrogen/progesterone receptor status		[150]
CXCL12 and CXCL16	Brain metastases-associated fibroblasts express significantly higher levels of CXCL12 and CXCL16 than fibroblasts from primary tumors or normal breast tissue, increasing tumor cell migration. Inhibition of CXCR4 or CXCL16 reduces tumor cell migration towards brain metastases-associated fibroblasts		[147]
IL8, IL1β, CXCL1, and HGF	Secretion of IL8 and CXCL1 is induced by c-Met signaling resulting from tumor cell adhesion to the brain endothelium. Additional IL1β secretion causes the release of HGF by astrocytes creating a feed-forward loop c-Met/HGF. Inhibition of c-Met inhibits brain metastases in vivo*.* IL1β is able to disrupt BBB integrity and is able to enhance tumor cell migration. Astrocytes are activated by brain metastatic tumor cells expressing IL1β, upregulating the expression of Notch ligand, which increases cancer stem-like cell proliferation. A Notch inhibitor significantly reduced metastatic outgrowth in vivo		[142, 146, 153]
IL6 and CCL2	Tumor cell overexpression of astrocytic sphingosine-1 phosphate receptor 3 (S1P3) enhances IL6 and CCL2 production by astrocytes, increasing BBB permeability. Inhibition of S1P3 significantly reduces disruption of the BBB both in vitro and in vivo		[148]
CCL5	In this triple-negative breast cancer study, microglia adopt a M2 phenotype in response to estrogen, reducing anti-tumor immune functions. Metastasis outgrowth is stimulated by microglia-secreted CCL5 in response to estrogen. Tamoxifen treatment and ovariectomy reduces microglial polarization and brain metastatic outgrowth in vivo		[152]
CXCL10	Cxcl10 mediates recruitment of VISTA^Hi^/PD-L1^+^ immune-suppressive CNS-native myeloid cells to brain metastatic tumors. Antibody blockage of VISTA and PD-L1 signaling reduces tumor outgrowth in vivo		[151]
NT-3	NT-3 mRNA levels significantly higher and NGF, BDNF and NT-4/5 mRNA levels significantly lower in brain metastatic tumor cells. In EMT-like tumor cells, ectopic NT-3 expression reduces migratory ability and increases HER2 and E-cadherin expression. The number of fully activated cytotoxic microglia is reduced by the endogenous and ectopic expression of NT-3		[172]
TGFβ2, TNF, and IL1β	Astrocytes produce TGF-β2 in response to tumor cell secreted IL-1β and TNF-α, upregulating Angiopoietin-like 4. Knockdown of Angiopoietin-like 4 reduces tumor cell outgrowth and improves survival in vivo		[169]
Angiopoietin-2	Angiopoietin-2 expression increased in tumor cells, associated with increased TJ disruption, and increased BBB permeability. Inhibiting angiopoietin-2 prevents BBB disruption and inhibits metastases formation in vivo		[143]
CXCL1, IL6, IL8, CSF-2, and CCL5	Tumor secretome upregulation in CXCL1, ICAM-1, IL6, IL8, CSF-2, and CCL5 compared to syndecan-1-silenced cells. Silencing of syndecan-1 significantly reduces brain metastases in vivo, while overexpressing increases metastases formation		[149]
IL6	Brain metastatic tumor cells induce immunosuppression via IL6 influence on programmed death-ligand-1 expressing myeloid cells. Tumor-related IL6 is also induces M2 microglia via JAK2/STAT3 signaling, promoting brain colonization	Lung cancer	[157, 158]
IGF-1 and CCL20	Nicotine enhances brain metastases by inducing M2 microglia phenotype, which increases the secretion of IGF-1 and CCL20, promoting metastatic outgrowth. Blocking M2 polarization reduces brain metastases in vivo		[159]
CSF-3	In brain metastatic tumor cells, pY696-EZH2-driven release of CSF-3 stimulated the recruitment of immunosuppressive neutrophils, which enhanced metastatic outgrowth. Anti-CSF-3 antibodies or immune checkpoint blockade therapies combined with Src inhibitors reduced tumor outgrowth in vivo		[162]
VEGF-A and TNF-α	Tumor cell secreted factors, including VEGF-A and TNF-α, increase E-selectin expression and damage the glycocalyx on cerebral endothelial cells in vitro. Endothelial glycocalyx degradation correlates with increased tumor cell adhesion. Inhibition of E-selectin attenuates tumor cell adhesion		[155]
MIF, IL8, IL6, TNF, and IL1β	Tumor cell factors, including MIF, IL-8 and plasminogen activator inhibitor-1 (PAI-1), activate astrocytes in vitro*.* Activated astrocytes secrete IL-6, TNF-α and IL-1β, promoting tumor cell proliferation		[154]
TNF	Increased expression of TNF facilitates E-selectin adhesion of tumor cells to brain endothelium via CD15		[156]
TGF-β	PREP1 modulates tumor cell sensitivity to TGF-β and is involved in EMT, facilitating metastases. Accumulation of PREP1 detected in human brain metastatic lesions		[161]
HGF	Co-overexpressing HGF and its receptor Met produces increased metastases, including to the brain		[144]
CCL17, CCL2, CXCL10, IL6, and IL1β	Upregulation of CCL17, CCL2, CXCL10, IL6, and IL-1β are associated with astrogliosis in the early stages of the metastatic cascade. CCL17 is also upregulated in vemurafenib-resistant melanoma cells in vivo	Melanoma	[163, 164]
TNF	TNF and MMP2 expression is associated with tumor cell aggressiveness. TNF inhibition reduces proliferation rate in 3 out of 4 tumor cell lines with the highly aggressive A375 cell line showing lower sensitivity to inhibition		[124]
TGF-β2	TGF-β2 is a site-specific growth factor for the brain parenchyma but not for the leptomeninges and ventricles		[165]
CXCL10	CXCL10 is upregulated in tumor-associated astrocytes in vivo, enhancing tumor cell migration toward astrocytes. The receptor for CXCL10, CXCR3 is increased in neurotropic tumor cells. Inhibiting CXCR3 expression reduces the formation of brain metastases in vivo		[166]
IL23	IL23 is highly expressed by metastases-associated astrocytes in vivo, increasing tumor cell MMP2 secretion and invasiveness. Knocking down MMP2 or blocking IL23 halted this increase in tumor invasion		[167]
CCL7	CCR2 and CCL7 expression was significantly greater in brain metastatic tumor cells compared with primary tumor cells	Renal cell cancer	[168]
CCL23, CXCL5, CXCL8, CCL8, CCL13, CCL17, and CCL18	In brain metastases, tumor-associated immune cells releases chemokines, including microglia (CXCL5 and CXCL8), monocyte-derived macrophages (CCL8, CCL13, CCL17, and CCL18) and tumor-associated macrophages (CCL23)	Multiple types	[247]
CCL2	Astrocyte-expressed CCL2 promotes tumor cell chemotaxis and chemokinesis without disrupting the BBB in vitro and in vivo. CCR2-deficient tumor cells show significantly reduced arrest and extravasation in vivo		[170]
MIF	PhosphoSTAT3 + reactive astrocytes associated with brain metastatic tumors reduce CD8 + T-cell activity and increase CD74 + microglia/macrophages population via a MIF–CD74–midkine axis, supporting tumor immune evasion		[171]
VEGF	Tumor cell expression of the VEGF mRNA and protein positively correlates with angiogenesis and growth of brain metastases		[248]

### Endothelial and tumor cell-surface molecules

Cell-surface adhesion molecules are involved in establishing tumor cell–EC interactions, and thus play an important role in arresting CTC in the cerebral microvasculature and facilitating extravasation across the BBB. Indeed, one of the first aspects of brain colonization is the adhesion of CTCs to brain ECs. While the exact steps and adhesion molecules involved are not well understood, a core feature of this process involves tumor cells mimicking aspects of the inflammatory leukocyte adhesion cascade, including arrest, adhesion and diapedesis [173, 174]. In addition to promoting extravasation, cell-surface molecules also play a diverse role in interactions with the brain’s resident cells and ECM, allowing for the formation of a favorable metastatic niche. Numerous cell-surface molecules located on the tumor cell or endothelial surface are implicated in the formation and outgrowth of brain metastases (Table 4). These include ALCAM [175–177], PECAM1 [178], L1CAM [179, 180], melanotransferrin [181], E-Selectin [155, 156, 182–186], and E-cadherin/ N-cadherin [187–190].

**Table 4:** Cell surface molecules associated with tumor cell extravasation across the blood–brain barrier and the formation of brain metastases

Cell surface molecules	Findings	Primary tumor	Refs
Integrins	Both in vivo and in human brain lesion specimens, tumor micrometastases localize to the neurovascular BL and co-opt existing vessels. Blockade of β_1_ integrin-mediated adhesion in tumor cells prevents adhesion to the neurovascular BL and decreases metastatic colonization and outgrowth in vivo	Breast cancer	[191]
	In vitro and in vivo activation of α_v_β_3_ causes the continuous post-transcriptional upregulation of VEGF, promoting the growth of metastatic brain lesions, but not the growth of the primary tumor		[192]
	Invasiveness of brain metastatic tumor cells influenced by the combined effects of α_v_ integrin and HER2, with α_v_ knockdown disrupting HER2 localization and reduced tumor cell motility in vitro and decreased brain invasiveness in vivo		[193]
	The anti-α_v_ monoclonal antibody, intetumumab, decreases brain metastases and increase survival in an in vivo animal model		[194]
	Increased β_4_ signaling disrupts brain EC junctional complexes by inducing HER2-dependent expression of VEGF		[195]
	Increased expression level of α_4_ß_1_ in brain metastatic tumor cells, both in vitro and in vivo. Receptor for α_4_ß_1_, VCAM1, widely expressed on the EC surface and as early as 5 days after intracardiac induction in vivo*.* Blockage of the α_4_ subunit significantly reduced in vivo metastatic seeding		[175]
	Rab11b-mediated cellular recycling of integrin β_1_ regulates brain metastatic breast cancer outgrowth, modulating interaction with ECM, facilitating mechanotransduction-activated survival signaling		[196]
	αB-crystallin expression in primary tumor associated with poor survival after brain metastasis. Overexpression of αB-crystallin enhances—and silencing inhibits—adhesion of tumor cells to ECs in vitro. Mechanism of adhesion partially achieved through α_3_β_1_ integrin. Brain metastases in vivo were increased or reduced by overexpressing or silencing αB-crystallin, respectively		[197]
	The antipsychotic agent, penfluridol, reduces the expression of integrin α_6_ and integrin β_4_ on tumor cells in vitro*.* Penfluridol treatment significantly inhibited the growth of brain metastases in vivo. Penfluridol-treated tumors demonstrated decreased integrin β_4_ and increased apoptosis		[198]
	High expression of α_v_β_5_ on vascular structures and tumor tissue in brain lesions associated with high hypoxia inducible factor 1α (HIF)-1α indices (related to tumor survival in hypoxic conditions). Brain lesions with a α_v_β_3_ expression pattern correlated with low Ki-67 proliferation indices and favorable survival times	Lung cancer	[201]
	Tumor cells with greater expression of α_3_ demonstrated greater ECM attachment, migration, and proliferation in vitro. Blocking α_3_β_1_ in vivo significantly decreases brain metastasis		[202]
	Over 90% of tested patient brain metastases expressed α_4_β_1._ In vitro antibody ablation of α_4_β_1_ reduces tumor cells arrest and BBB disruption	Melanoma	[203]
	Expression of α_v_β_6_ significantly higher in brain metastases with well-demarcated growth compared to vascular co-option and diffuse infiltration. Expression of α_v_ in patient brain metastatic lesions significantly higher than in the primary tumor	Multiple types	[199, 200]
ALCAM	High tumor ALCAM expression and increased ALCAM endothelial expression in vivo during early metastasis seeding. Anti-ALCAM antibodies significantly decreased brain metastasis seeding in vivo	Breast cancer	[175]
	ALCAM expression significantly increased in patient brain metastases, with increased expression in primary tumor and brain metastases associated with shortened survival. In vitro ALCAM knockdown reduces tumor cell adhesion to cerebral endothelial cells. ALCAM knockdown tumor cells produced reduced brain metastatic tumor seeding in vivo	Lung cancer	[176]
	Proof-of-concept study for ALCAM-targeting MRI contrast agent using in vivo brain metastasis model. ALCAM-targeting contrast agent was able to detect brain micrometastases from lung, breast and skin cancer	Multiple types	[177]
VCAM1	VCAM1 is expressed in human brain metastases and micrometastases. Targeted MRI contrast agent for VCAM1 revealed upregulated expression in brain metastases 5 days after induction in vivo	Breast cancer	[210]
	Induction of cerebrovascular inflammation significantly increases brain expression of VCAM1 in vivo*.* Intracardiac injection of tumor cells in mice with induced cerebrovascular inflammation increases brain metastatic burden, however blocking VCAM1 before tumor cell injection prevents this increase		[208]
	Anti-VCAM1 antibody produces significant reduction in brain metastatic burden and increased overall survival in vivo		[211]
	Increased VCAM1 expression and microvessel density at the boundary of tumor tissue and surrounding brain tissue in animal xenograft model. Similar results were observed in human brain metastasis specimens		[209]
	Anti-VCAM1 antibodies partly inhibit tumor cells adhesion to brain ECs	Prostate cancer	[185]
	VCAM-1 expression in human brain metastasis specimens, across lung, breast and skin cancer. Targeted MRI contrast agent for VCAM1 revealed upregulated VCAM1 in tumor-associated microvessels	Multiple types	[207]
PECAM1	PECAM1 associated with a highly brain metastatic tumor cell model	Breast cancer	[178]
L1CAM	L1CAM mediates vascular co-option by brain metastases, which is promoted by serpins. L1CAM also activates YAP via integrin β_1_ and integrin-linked kinase, facilitating metastatic outgrowth	Multiple types	[179, 180]
Melano-transferrin	Tumor cell ability to extravasate across the BBB correlates with tumor cell-surface expression of melanotransferrin in vitro. Application of anti-melanotransferrin antibody significantly reduced the development of brain metastases in vivo	Melanoma	[181]
E-Selectin	E-selectin promotes adhesion and extravasation of estrogen receptor^(–)^/CD44^(+)^ tumor cells, but not estrogen receptor^(+)^/CD44^(−/low)^ tumor cells in vitro. In estrogen receptor^(–)^ breast cancer, CD44^(+)^ tumor cells are found in high quantities in human brain lesion specimens. In vivo application of an E-selectin antagonist significantly reduced brain metastases	Breast cancer	[182]
	Concomitant high expression of BST-2 with CD15s (E-Selectin binding partner) in ER-negative tumors from patients is associated with higher risks of liver and brain metastasis and decreased survival rate		[183]
	Tumor cells and tumor-secreted factors increase E-selectin expression on cerebral endothelial cells in vitro. Endothelial glycocalyx degradation correlates with increased tumor cell adhesion. Inhibition of E-selectin attenuates tumor cell adhesion	Lung cancer	[155]
	TNF-α associated with increased E-selectin on cerebral ECs. Immunoblocking of the E-selectin binding partner CD15 on tumor cells reduces adhesion to cerebral ECs. Both CD15 and E-selectin are expressed in patient brain metastatic lesions. Overexpression of CD15/CD15s increases tumor cell adhesion to the E-selectin on cerebral ECs, increasing the disruption of cerebral endothelial cell monolayers. Knockdown of FUT4/ FUT7, which code for CD15/CD15s, prevents in vitro BBB disruption. Overexpression of FUT4/ FUT7 in non-metastatic tumor cells increases metastatic phenotype		[156, 184]
	Tumor cell adhesion significantly increased by upregulated TNF-α, with increased E-selectin expression on cerebral ECs. Anti-E-selectin antibodies partly inhibit adhesion of tumor cells to brain ECs. Human tumor cells derived from brain metastases express the E-selectin ligand, E-selectin ligand-1	Prostate cancer	[185, 186]
E-cadherin/ N-cadherin	Increased E-cadherin expression in metastases to the liver, lung and brain compared to the primary tumor. Ectopic expression of E-cadherin causes tumor cells with mesenchymal phenotype to revert to epithelial phenotype in vitro. Tumor cells with a mesenchymal phenotype injected into primary tumor site express E-cadherin after metastasizing in vivo. E-cadherin linked to resistance to ionizing radiation and chemotherapy	Breast cancer	[187, 190]
	N-cadherin expression highly predictive of brain metastasis–free survival. Low E-cadherin expression in patients associated with increased risk of developing brain metastasis	Lung cancer	[188, 189]

The integrin family of adhesion molecules are shown to play a particularly important role in establishing brain colonization across breast cancer [175, 191–200], lung cancer [201, 202] and melanoma [203]. For example, multiple studies implicate the integrin subunit α_v_ in brain metastatic breast cancer, with human brain lesion specimens exhibiting significant expression of α_v_ integrins [200]. Moreover, this α_v_ expression is significantly higher in brain lesion specimens than in the primary tumor. In addition, the anti-integrin α_v_ monoclonal antibody, intetumumab, is shown to decrease breast cancer brain metastases and increase survival in vivo [194]. Integrins may also allow CTCs to indirectly use platelets and leukocytes as a means of bridging the endothelium, overcoming the low expression of adhesion molecules on the neurovascular EC surface [204, 205]. For example, upregulation of α_v_β_ɜ_ integrin facilitates tumor cell interaction with platelets in vivo causing thrombus formation, which promotes arrest of CTCs in the blood vessel, as well as metastatic progression [206]. Integrins may additionally play a signal transduction role in brain metastases, along with adhesion functions. For instance, increased signaling by β_4_ integrin in breast cancer CTCs was shown to disrupt brain EC junctional complexes by inducing HER2-dependent expression of VEGF [195].

Beyond ECs, Carbonell and colleagues demonstrate that breast cancer micrometastases, both in vivo and in human brain lesion specimens, localize to the neurovascular BL, with integrin β_1_ implicated in this process. Blockade of β_1_ integrin-mediated ECM adhesion in tumor cells prevented binding to the neurovascular BL, decreasing metastatic colonization and outgrowth in vivo [191]. VCAM1, the binding partner of integrin α_4_ß_1_, is also strongly implicated in the development of brain metastases, with endothelial expression significantly upregulated early on in metastatic seeding and in the tumor-associated microvessels [207–209]. Recent work highlights the potential of VCAM1 as a highly sensitive diagnostic marker for brain metastases using MRI [207, 209, 210], while anti-VCAM1 antibodies are a promising avenue of treatment [185, 208, 211].

Finally, tumor cell adhesion to the endothelium not only requires the appropriate surface molecules to be present, but also that these molecules are in a receptive state for cell–cell interactions. Brain metastatic breast cancer cells are shown have enriched expression of the gene ST6GALNAC5, which encodes for α-2,6-sialyltranserase [120]. Normally exclusive to the brain, this sialyltransferase catalyzes the addition of sialic acid to cell-surface glycoproteins. In turn, this alteration to surface glycoproteins is able to stimulate tumor cell adhesion, migration, and invasion [120, 212]. Taken together, numerous cancers associated with brain metastases are associated with the increased expression of cell-surface adhesion molecules. These molecules, in turn, support interaction with the cerebral endothelium, the arrest of tumor cells in the brain, extravasation across the BBB and manipulation of the neurovascular niche.

## Malignancies at the neurovascular niche: brain metastatic and glioma progression along the blood–brain barrier

The neurovascular niche plays a crucial role in the progression of brain metastases once CTCs have crossed the BBB. For DTC to survive after extravasation, they require continuous contact with the ECs and ECM associated with brain microvessels. Indeed, numerous studies now show that DTC attach to the abluminal BL, providing access to oxygen and metabolites, as well as positioning these tumor cells for vessel co-option and metastatic outgrowth [10, 84, 179, 191]. Indeed, this appears to be an important element in the initial survival of DTCs, as early-stage migration beyond the neurovascular niche into the brain parenchyma appears universally fatal [10]. An important additional aspect of metastatic development in the brain is the ability of DTCs to remain dormant in the perivascular microenvironment as micrometastases, sometimes for many years [213]. Recent work shows that multiple aspects of the BBB are involved in the perivascular dormancy of DTCs, including endothelial-derived thrombospondin-1 and astrocyte-deposited laminin-211 [214, 215].

While the precise mechanisms that awaken dormant micrometastases and activate tumor outgrowth remain unresolved, once outgrowth is underway metastatic progression can take on various invasion patterns. Interestingly, the primary tumor does not appear to be a strong predictor of which invasion pattern is observed [199]. The most prominent patterns of macrometastatic outgrowth in the brain are: (i) a displacing or non-infiltrating outgrowth pattern, producing a clear tumor boundary without infiltration into the adjacent tissue, (ii) a diffuse invasion, involving either single cells or small macrometastatic outgrowths infiltrating the brain parenchyma, and (iii) angiotropic invasion or vessel co-option, in which tumor cells sheath along adjacent blood vessels, protruding into the brain tissue [199, 216]. Here, we focus on the latter of these invasion patterns and discuss the mechanisms promoting brain metastatic vessel co-option. Interestingly, the role of the neurovascular niche in malignant outgrowth in the brain is not limited to metastases, as glioma, the most frequent primary brain tumor, can also adopt an invasive pattern modeled along the brain’s blood vessels. Because of this commonality, we also highlight the mechanisms facilitating vessel co-option in glioma and discuss possible implications for the treatment of brain malignancies.

Tumor angiogenesis involves tumor cells in the brain stimulating the sprouting and proliferation of new vessels to support a microenvironment favoring malignant outgrowth. Vessel co-option in the brain is defined as a non-angiogenic process in which tumor cells hijack existing blood vessels, either (i) by ensheathing pre-existing vessels without widespread disruption to the BBB, or (ii) by displacing pericytes and astrocytes, manipulating ECs, and remodeling the neurovascular ECM, often causing BBB disruption. In addition to these direct, perivascular forms of vessel co-option, tumor cells are also able to move in a diffuse, vascular adjacent manner through the brain parenchyma. These methods of co-opting pre-existing vessels aid in tumor cell survival, providing access to nutrients and oxygen, as well as supplying a ready-built path to further invasion. Vessel co-option is also implicated in circumvention of the BBB, as acute lymphoblastic leukemia cells have been shown to co-opt and transit along bridging vessels (facilitated by integrin α_6_) and enter the cerebral spinal fluid without having to extravasate into the brain [217].

Preclinical evidence indicates that across brain metastatic breast, lung, skin and colorectal cancer cell lines, vessel co-option is a frequent and important contributor to tumor vascularization and metastatic outgrowth, often together with angiogenesis [10, 123, 218, 219]. Interestingly, similar findings are observed for low-grade glioma, with vessel co-option being an important feature of early malignant outgrowth [220–223]. In contrast, tumor angiogenesis is widely understood to play a prominent role in disease progression in the high-grade, aggressive glioma variant glioblastoma multiforme (GBM), including hyper-vascularization, endothelial proliferation and blood vessel malformation [224, 225]. This has led to a broad focus on developing anti-angiogenic treatments that target the pathological mechanisms (e.g., enhanced VEGF signaling) facilitating blood vessel growth. However, even at this late stage of the disease, preclinical studies suggest that vessel co-option still occurs [223, 226]. Findings from human brain lesion specimens support this preclinical evidence in both brain metastases and in glioma. In brain metastases, clinical specimens from brain metastatic breast, lung, skin and colorectal cancer have shown the occurrence of vessel co-option [10, 191, 199, 216, 227]. Similarly, vessel co-option has been observed in human GBM lesions [226, 228]. However, despite this preclinical and clinical evidence for vessel co-option in both brain metastases and glioma, no comprehensive study has established the relative occurrence of vessel co-option and angiogenesis in either of these brain malignancies [229].

With respect to mechanism, vessel co-option in brain metastases has been shown to involve cell adhesion molecules on the tumor cell surface, including integrin β_1_ [191] and L1CAM [179, 180] establishing attachment and sheath formation along the abluminal vessel surface and creating an integrated growth front that uses the existing vasculature. With regards to GBM, several factors are implicated in promoting vessel co-option, including bradykinin [230], Mammary-derived growth inhibitor [231], CXCR4 [232], EGFRvIII [233], Angiopoietin-2 [222], IL8 [234, 235], IRE-1α [236], and Wnt7 [237]. Moreover, GBM vessel co-option has been shown to either involve complete ensheathment of the vessel without significant alteration to the BBB or direct attachment to the abluminal surface of EC, disrupting the BBB and displacing both astrocytes and pericytes. Indeed, in this latter instance GBM has been shown to adopt the attributes of pericytes and in doing so influence the function of the surrounding neurovasculature in a process termed pericyte mimicry [238]. Interestingly, pericyte-like spreading along blood vessels is also observed in brain metastases [180].

With regards to therapy, over the past several decades a strong focus has been placed on the development of anti-angiogenic drugs (such as VEGF inhibitors) to treat both brain metastases and glioma [239, 240]. However, a growing body of evidence indicates that anti-angiogenic therapy, although often initially successful in reducing tumor size and growth, creates a selective environment that can induce a shift to the vessel co-option invasion pattern [241]. This process, in turn, may facilitate the often observed therapeutic resistance to anti-angiogenic drugs in both types of brain malignancy. However, to date, no clear consensus exists regarding the exact role of vessel co-option in the development of anti-angiogenic therapy resistance. Thus, illuminating the mechanisms of anti-angiogenic resistance and the potential involvement of vessel co-option is an important strand of preclinical research. Indeed, the study of vessel co-option in brain malignancies is an emerging field and anti-vessel co-option therapies may prove to be an important treatment option, complementary to anti-angiogenic therapies for both glioma and for brain metastases. Moreover, an integrated understanding of the means by which glioma and brain metastases interact with the perivascular microenvironment can inform the development of such treatments. Ultimately, improvements in detecting vessel co-option, along with a better understanding of the underlying mechanisms may lead to the development of interventions that mitigate this form of invasive outgrowth in the brain.

## Conclusion and perspective

Despite improvements in extracranial therapy, survival rates for patients suffering from brain metastases remain very poor. This is coupled with the fact that the incidence of brain metastases is continuing to rise. The evidence discussed in the current work indicates that metastatic brain invasion involves neurotropic mechanisms, which facilitate BBB disruption and crossing. Moreover, the neurovascular niche is demonstrated to play an important role in supporting brain malignancies, with vessel co-option an important step in promoting tumor outgrowth for both brain metastases and glioma. The neurotropic toolkit that metastatic tumor cells are able to develop involves a range of genetic alterations, secreted/shredded factors, and surface molecules, which manipulate the NVU, forming a neurovascular metastatic niche. This is mediated partially by altering the normal physiology of resident cells and remodeling the associated ECM to create a perivascular metastatic microenvironment favorable for tumor cell survival. Given that brain metastases are a frequent and deadly consequence of advanced peripheral primary tumors, the development of clinical methods to detect and target the neurotropic mechanisms underlying metastatic brain invasion are of clear practical importance.

In particular, preventive and predictive approaches to the management of brain metastases present a promising avenue of future research. With regard to preventing brain metastases, one current approach involves prophylactic intercranial radiation for cancers including non-small-cell and small-cell lung cancers [242]. However, despite some efficacy in this means of prevention, the profound negative sequelae following this approach, broadly termed radiation-induced cognitive dysfunction, limit the application of this method. In contrast, developing a better understanding, identification, and characterization of the neurotropic changes occurring in the primary tumor and CTCs may allow for a targeted approach to the prevention of brain metastases, both for initial lesion occurrence and for relapse after surgical resection. For example, a recent phase 3 trial in ALK-positive non-small-cell lung cancer showed that the use of the ALK tyrosine kinase inhibitor alectinib was associated with longer progression-free survival and worked activity against the development of brain metastases [243, 244]. Similarly, the EGFR tyrosine kinase inhibitor Lapatinib has shown promise in preventing lesion relapse in HER2-positive breast cancer [245]. In addition, immunotherapies aimed at targeting neurotropic chemokine axes in brain metastases may also represent a promising means of preventing brain metastases [246].

In a similar vein, methods to predict the likelihood or onset of brain metastases represent a further an avenue of valuable research, able to support methods of prevention. For example, several recent studies have examined the pattern of MiR in patients’ serum or primary tumor as a means of creating a brain metastases-specific biomarkers [136, 138]. Such biomarkers could, in turn, inform targeted prevention, treatment, and management. Ultimately, our ability to predict, prevent, and treat brain metastases is limited by the complex and adaptive heterogeneity of both the primary and secondary tumors. Furthering our early-stage understanding of the neurotropic mechanisms available to cancer cells may allow for targeted approaches that are able overcome the heterogeneity of these malignancies.

In conclusion, a growing body of evidence suggests that neurotropic mechanisms can facilitate the formation of both a pre-metastatic and metastatic niche at the BBB. Basic research is required to gain a better understanding of such events. This research, in turn, could support the development of preemptive interventions to mitigate brain metastases. Furthermore, accurate detection and interpretation of neurotropic patterns associated with CTCs in patients with extracranial malignancies may improve the diagnosis and prognosis of brain metastases, as well as inform early treatment. At the same time, understanding the perivascular conditions that promote the dormancy of micrometastases at the BBB, as well as the early-stage events that awaken and activate macrometastatic outgrowth is an essential avenue of research. Therapeutics that are able to maintain tumor cell dormancy may offer a long-term means of controlling the malignant outgrowth of micrometastases in the brain. Finally, a better understanding of the mechanisms underlying invasion in the perivascular microenvironment, including the potential of tumor cells to hijack existing vasculature, offer the potential to develop novel anti-vessel co-option therapies that could aid in the treatment of malignancies of the brain.

## Data Availability

Not applicable.
